# Supplementary material for: Heat stress illness outcomes and annual indices of outdoor heat at U.S. Army installations
Source: PLoS One. 2022 Nov 23;17(11):e0263803. doi: 10.1371/journal.pone.0263803 (PMC9683623; doi:10.1371/journal.pone.0263803)
Supplement: Attachment — Submitted filename: PONE-D-22-02473_reviewer.pdf [file pone.0263803.s001.pdf]

PLOS ONE

Heat Stress Illness Outcomes and Annual Indices of Outdoor Heat at U.S. Army Installations

--Manuscript Draft--

**Manuscript Number:**	PONE-D-22-02473
**Article Type:**	Research Article
**Full Title:**	Heat Stress Illness Outcomes and Annual Indices of Outdoor Heat at U.S. Army Installations
**Short Title:**	Heat Stress Illness Outcomes and Annual Indices of Outdoor Heat at U.S. Army Installations
**Corresponding Author:**	Stephen Archie Lewandowski, MPhil, SM<br>Uniformed Services University of the Health Sciences<br>Bethesda, MD UNITED STATES
**Keywords:**	climate; heat stress; heat stroke; heat exhaustion; military; environmental health
**Abstract:**	This study characterized associations between annually scaled thermal indices and annual heat stress illness (HSI) morbidity outcomes, including heat stroke and heat exhaustion, among active-duty soldiers at ten Continental U.S. (CONUS) Army installations from 1991 to 2018. We fit negative binomial models for 3 types of HSI morbidity outcomes and 104 annual indices, adjusting for installation-level effects and long-term trends with a block bootstrap approach. Ambulatory (out-patient) and reportable event HSI outcomes displayed positive association patterns with the assessed annual indices of heat in our models. For example, a one-degree Fahrenheit (°F) increase in mean temperature between May and September was associated with a 1.05 (95% confidence interval [CI]: 1.00, 1.11) times greater rate of ambulatory encounters. The annual-scaled rate ratios and their uncertainties may be applied to climate projections for a wide range of thermal indices to estimate future HSI burden and impacts to medical readiness.
**Order of Authors:**	Stephen Archie Lewandowski, MPhil, SM
	Marianthi-Anna Kioumourtzoglou, ScD
	Jeffrey L. Shaman, PhD
**Additional Information:**	
**Question**	**Response**
**Financial Disclosure**<br><br>Enter a financial disclosure statement that describes the sources of funding for the work included in this submission. Review the submission guidelines for detailed requirements. View published research articles from *PLOS ONE* for specific examples.<br><br>This statement is required for submission and **will appear in the published article** if the submission is accepted. Please make sure it is accurate.	The opinions and assertions expressed herein are those of the authors and do not necessarily reflect the official policy or position of the Uniformed Services University or the Department of Defense. S.L was supported by the U.S. Army Long Term Health Education and Training (LTHET) program. J.S. and Columbia University disclose partial ownership of SK Analytics. J.S. also reports receiving consulting fees from Merck and BNI. The funders had no role in study design, data collection and analysis, decision to publish, or preparation of the manuscript.

Powered by Editorial Manager® and ProduXion Manager® from Aries Systems Corporation

**Unfunded studies**<br>Enter: *The author(s) received no specific funding for this work.*<br><br>**Funded studies**<br>Enter a statement with the following details:<br>• Initials of the authors who received each award<br>• Grant numbers awarded to each author<br>• The full name of each funder<br>• URL of each funder website<br>• Did the sponsors or funders play any role in the study design, data collection and analysis, decision to publish, or preparation of the manuscript?<br>• **NO** - Include this sentence at the end of your statement: *The funders had no role in study design, data collection and analysis, decision to publish, or preparation of the manuscript.*<br>• **YES** - Specify the role(s) played.<br><br>* typeset	
**Competing Interests**<br><br>Use the instructions below to enter a competing interest statement for this submission. On behalf of all authors, disclose any competing interests that could be perceived to bias this work—acknowledging all financial support and any other relevant financial or non-financial competing interests.<br><br>This statement is required for submission and **will appear in the published article** if the submission is accepted. Please make sure it is accurate and that any funding sources listed in your Funding Information later in the submission form are also declared in your Financial Disclosure statement.<br><br>View published research articles from *PLOS ONE* for specific examples.	The authors have declared that no competing interests exist.

**NO authors have competing interests**<br><br>Enter: *The authors have declared that no competing interests exist.*<br><br>**Authors with competing interests**<br><br>Enter competing interest details beginning with this statement:<br><br>*I have read the journal's policy and the authors of this manuscript have the following competing interests: [insert competing interests here]*<br><br>* typeset	
**Ethics Statement**<br><br>Enter an ethics statement for this submission. This statement is required if the study involved:<br><br>• Human participants<br>• Human specimens or tissue<br>• Vertebrate animals or cephalopods<br>• Vertebrate embryos or tissues<br>• Field research<br><br>Write "N/A" if the submission does not require an ethics statement.<br><br>General guidance is provided below. Consult the submission guidelines for detailed instructions. **Make sure that all information entered here is included in the Methods section of the manuscript.**	N/A

Powered by Editorial Manager® and ProduXion Manager® from Aries Systems Corporation

**Format for specific study types**<br><br>**Human Subject Research (involving human participants and/or tissue)**<br>• Give the name of the institutional review board or ethics committee that approved the study<br>• Include the approval number and/or a statement indicating approval of this research<br>• Indicate the form of consent obtained (written/oral) or the reason that consent was not obtained (e.g. the data were analyzed anonymously)<br><br>**Animal Research (involving vertebrate animals, embryos or tissues)**<br>• Provide the name of the Institutional Animal Care and Use Committee (IACUC) or other relevant ethics board that reviewed the study protocol, and indicate whether they approved this research or granted a formal waiver of ethical approval<br>• Include an approval number if one was obtained<br>• If the study involved *non-human primates*, add *additional details* about animal welfare and steps taken to ameliorate suffering<br>• If anesthesia, euthanasia, or any kind of animal sacrifice is part of the study, include briefly which substances and/or methods were applied<br><br>**Field Research**<br><br>Include the following details if this study involves the collection of plant, animal, or other materials from a natural setting:<br>• Field permit number<br>• Name of the institution or relevant body that granted permission	
**Data Availability**<br><br>Authors are required to make all data underlying the findings described fully available, without restriction, and from the time of publication. PLOS allows rare exceptions to address legal and ethical concerns. See the PLOS Data Policy and FAQ for detailed information.	No - some restrictions will apply

A Data Availability Statement describing where the data can be found is required at submission. Your answers to this question constitute the Data Availability Statement and **will be published in the article**, if accepted.<br><br>**Important:** Stating 'data available on request from the author' is not sufficient. If your data are only available upon request, select 'No' for the first question and explain your exceptional situation in the text box.<br><br>Do the authors confirm that all data underlying the findings described in their manuscript are fully available without restriction?	
**Describe where the data may be found in full sentences. If you are copying our sample text, replace any instances of XXX with the appropriate details.**<br><br>• If the data are **held or will be held in a public repository**, include URLs, accession numbers or DOIs. If this information will only be available after acceptance, indicate this by ticking the box below. For example: *All XXX files are available from the XXX database (accession number(s) XXX, XXX.)*.<br>• If the data are all contained **within the manuscript and/or Supporting Information files**, enter the following: *All relevant data are within the manuscript and its Supporting Information files.*<br>• If neither of these applies but you are able to provide **details of access elsewhere**, with or without limitations, please do so. For example:<br><br>*Data cannot be shared publicly because of [XXX]. Data are available from the XXX Institutional Data Access / Ethics Committee (contact via XXX) for researchers who meet the criteria for access to confidential data.*<br><br>*The data underlying the results presented in the study are available from (include the name of the third party*	Climate index data are available from https://doi.org/10.5281/zenodo.5903145. Outcome data are available from the Defense Medical Epidemiology Database (DMED) at https://www.health.mil/Military-Health-Topics/Combat-Support/Armed-Forces-Health-Surveillance-Division/Data-Management-and-Technical-Support/Defense-Medical-Epidemiology-Database. DMED is available to authorized users such as U.S. military medical providers, epidemiologists, medical researchers, safety officers or medical operations/ clinical support staff for surveying health conditions in the U.S. military. Civilian collaborators in military medical research and operations may also have access to DMED with documentation supporting their arrangements.

and contact information or URL).<br>• This text is appropriate if the data are owned by a third party and authors do not have permission to share the data.<br><br>* typeset	
Additional data availability information:	

Manuscript
Click here to access/download;Manuscript;annual_heat_manuscript_plos.docx

Heat Stress Illness Outcomes and Annual Indices of Outdoor Heat at U.S. Army Installations

Stephen A. Lewandowski[1,2, #a*], Marianthi-Anna Kioumourtzoglou[1], Jeffrey L. Shaman[1]

[1] Department of Environmental Health Sciences, Columbia University Mailman School of Public Health, New York, New York, United States of America

[2] Department of Preventive Medicine and Biostatistics, Uniformed Services University of the Health Sciences, Bethesda, Maryland, United States of America

[#a] Current Address: Department of Preventive Medicine and Biostatistics, Uniformed Services University of the Health Sciences, Bethesda, Maryland, United States of America

* Corresponding author

E-mail: stephen.lewandowski@usuhs.edu (SL)

Acknowledgements / Disclosures.

The opinions and assertions expressed herein are those of the authors and do not necessarily reflect the official policy or position of the Uniformed Services University or the Department of Defense. S.L was supported by the U.S. Army Long Term Health Education and Training (LTHET) program. J.S. and Columbia University disclose partial ownership of SK Analytics. J.S. also reports receiving consulting fees from Merck and BNI.

Abstract

This study characterized associations between annually scaled thermal indices and annual heat stress illness (HSI) morbidity outcomes, including heat stroke and heat exhaustion, among active-duty soldiers at ten Continental U.S. (CONUS) Army installations from 1991 to 2018. We fit negative binomial models for 3 types of HSI morbidity outcomes and 104 annual indices, adjusting for installation-level effects and long-term trends with a block bootstrap approach. Ambulatory (out-patient) and reportable event HSI outcomes displayed positive association patterns with the assessed annual indices of heat in our models. For example, a one-degree Fahrenheit (°F) increase in mean temperature between May and September was associated with a 1.05 (95% confidence interval [CI]: 1.00, 1.11) times greater rate of ambulatory encounters. The annual-scaled rate ratios and their uncertainties may be applied to climate projections for a wide range of thermal indices to estimate future HSI burden and impacts to medical readiness.

Introduction

Heat stress illnesses (HSIs) pose a preventable, potentially fatal, health threat with serious impacts to military training and readiness [1,2]. HSIs occur when the effects of environmental heat stress, combined with metabolic heat generated from physical activity, exceed thermoregulatory and heat exchange capacities, resulting in elevated core temperature [3]. This heat strain manifests as a continuum of outcomes, including heat stroke, heat exhaustion, edema, cramps, and fainting. In the U.S. Arm[illegible]iagnosed cases of heat stroke and heat exhaustion have increased in recent years as average annual temperatures and high temperature records continue to rise [4,5]. Military service members who train in the Continental U.S. (CONUS) experience

elevated risks from heat exposure compared to similar age groups in the general population due to increased time outdoors, high physical exertion levels, clothing burden, and equipment loads.

The environmental properties affecting heat exchange include air temperature, air humidity, wind speed, and solar, sky, and ground radiation [3]. A wide range of methods and indices exist to classify the thermal environment as it relates to thermal stress and physiological effects [6]. The primary index used by the U.S. Army is the wet bulb globe temperature (WBGT). The WBGT is a weighted average of natural wet-bulb temperature (weight, *w*=70%), globe thermometer temperature (*w*=20%), and dry-bulb temperature (*w*=10%) in outdoor, non-shaded conditions [7]. Another commonly reported metric is the U.S. National Weather Service's (NWS) heat index. The NWS heat index (HI) represents an apparent temperature measure of thermal comfort based on air temperature and relative humidity and serves as a basis for excessive heat warnings [8]. The U.S. Army Public Health Center also applies the NWS HI as an indicator for heat risk days, defined as days with an HI greater than 90 degrees Fahrenheit (°F) for more than one hour [4]. Although the WBGT and HI are most often applied to short-term (hourly, daily, heat wave event) exposures, averages or aggregates from these instruments can also assist with characterization of long-term (seasonal, annual) heat and humidity risks.

The objective of this study was to characterize the association between indices of heat and annual HSI morbidity outcomes among active-duty soldiers at ten CONUS Army installations in the context of rising temperature and humidity conditions. Resulting estimates for the sensitivity of health outcomes to changing environmental conditions can inform long-term planning assumptions and provide a basis for evaluating the effectiveness of prevention measures.

Materials and Methods

Health Outcome Data

We obtained HSI outcome counts and rates of hospitalization (in-patient), ambulatory visits (out-patient), and reportable medical events from the Defense Medical Epidemiology Database (DMED), which contains summarized, non-Privacy Act data from the Defense Medical Surveillance System (DMSS) [9]. We queried primary diagnosis International Classification of Diseases (ICD) codes for active-duty U.S. Army servicemembers. For ICD-9, used through 2015, we applied 992-series codes, categorized as “effects of heat and light” [10]. We used ICD-10 series T67 codes for 2016 – 2018 data [11]. The counts and rates in this study aggregate all conditions within these code groups, with heat stroke and heat exhaustion representing the majority of cases across each of the three outcome types. Hospitalization data were available from 1990 – 2018, ambulatory from 1997 – 2018, and reportable events from 1995 – 2018. We excluded the initial years for hospitalizations and ambulatory encounters (1990 and 1997, respectively) from analyses due to signs of incomplete reporting. Additionally, we queried injuries and illnesses of all types to consider potential long-term trends due to changes in reporting systems or access-to-care and to assess the relative burden of disease due to HSIs. We selected ten U.S. Army CONUS installations based on previously reported *Medical Surveillance Monthly Report (MSMR)* HSI rates and exploratory DMED findings [1]. The ten included locations account for over 78% of all CONUS active-duty Army HSI cases for the examined years. Fort Irwin, CA, the next highest location, reported less than half the HSI cases as the tenth

ranked site, Fort Bliss, TX, and features rotational training cycles at its National Training Center that challenge exposure assumptions.

Meteorology Data

Meteorological estimates from the North American Land Data Assimilation System 2 (NLDAS-2) forcing dataset served as the primary source of weather and atmospheric data[12]. NLDAS is a National Aeronautics and Space Administration (NASA) / National Oceanic and Atmospheric Administration (NOAA)-led multi-institution project that constructs gridded surface meteorological datasets through the assimilation and merging of fields derived from gauge-based and remotely-sensed observations and re-analyses, with validation from ground-based observations [12]. NLDAS-2 data are available on a 1/8th-degree spatial scale at hourly frequencies from 1979 to present. We selected NLDAS grid cells containing the centroid of each installation based on shapefiles from the Department of Defense (DoD) Military Installations, Ranges, and Training Areas (MIRTA) Dataset [13].

NLDAS fields include air temperature at 2 meters above the surface, specific humidity at 2 meters above the surface, surface pressure, wind speed, and bias-corrected surface downward shortwave radiation. We calculated relative humidity from specific humidity, temperature, and atmospheric pressure; heat index (HI) from temperature and relative humidity based on a US National Weather Service algorithm[8]; and outdoor WBGT from air temperature, relative humidity, solar irradiance, barometric pressure, and wind speed using the method of Liljegren *et al.*, based on principles of heat and mass transfer [14,15]. We conducted sensitivity analyses comparing NLDAS centroid estimates with eleven years of hourly data produced by the 14th Weather Squadron from nearest weather station [illegible]d observed consistency in line with the distance to the station.

We compiled 104 annual indices of hea[illegible]rough multiple aggregations of hourly temperature, HI, and WBGT estimates in absolute and relative terms, averaged either over the entire calendar year or the heat season, defined as 01 May through 30 September. We included the former full-year average, as a prior evaluation assessed that approximately 17% of all HSI cases occurred during non-summer months, variable by location [16]. Absolute measures included annual mean temperatures and counts of heat risk days or hours above specified thresholds based on heat category cut-offs for HI and WBGT. Mean values were calculated over 24-hour periods to capture minimum temperatures, which can impact recovery from heat exposure. We calculated relative measures with reference to 1990 to 2019 climatologies for each day of the year and each location. These relative indices included annual mean daily anomalies, annual maximum daily anomalies, counts of days above daily 85th, 90th, and 95th percentiles (mean and maximum values), and counts of days one or two standard deviations above daily temperature climate norms for mean and maximum values.

Statistical Analyses

To evaluate time trends in our exposure metrics, we fit linear models regressing each index of heat on time for each installation. We evaluated outcome measures in a similar manner, with simple linear regressions for rates of each outcome type over time, by installation and with combined rates ($\frac{sum\ of\ counts}{sum\ of\ population}$) for all ten installations.

We applied negative binomial regression to model the over-dispersed count outcomes for hospitalizations, ambulatory encounters, and reportable events [17]. The index of heat served as the exposure of interest, in units of °F, number of days, or number of hours. We set indicator

variables for each installation to account for potentially confounding factors varying across installations and set the active-duty Army population of each installation for each year as an offset. Our resulting regression formula for the log of the rate predicted by the exposure and installation indicator variables is: $\log\left(\frac{\widehat{outcome\ count_i}}{population_i}\right) = \widehat{\beta_0} + \widehat{\beta_1} index\ value + \sum_{j=2}^{10} \widehat{\beta_j} I\ (installation_i = j)$, with Fort Bliss, TX set as the reference installation. We accounted for confounding by year, which is associated with long-term trends in both the exposure and outcome, by applying a block bootstrap approach that shuffles replicated selections of the data to reduce effects of serial correlation [18]. The time variable includes elements which we are limited in our ability to decompose, such as changes in access to care, admission protocols, coding practices, and reporting systems in addition to soldier demographics, fitness levels, and training intensities. We hypothesize that if we were to only include *year* as a term in a standard model without a blocked bootstrap approach, the trend would capture a portion of the outcome variability associated with the changes in heat we are investigating and bias estimates towards the null, while failure to adjust for trends through time in any manner would bias results away from the null.

To construct block bootstraps, we randomly selected two-year intervals with replacement and assembled these intervals into a new series with the approximate length of the base time series. We conducted 10,000 replications of this process on select indices for each model (represented in Fig 2), calculated beta coefficients for each iteration, and constructed nonparametric basic (empirical) bootstrap confidence intervals [19,20]. For the remainder of indices, we conducted 2,000 bootstrap replications. We assessed sensitivity by comparing non-bootstrap models (with and without a year term), original single observation bootstraps, and three-year block interval bootstraps. In the two-year block models, we also examined bias-

corrected and accelerated (BCa) bootstrap intervals, which incorporate parameters for the proportion of bootstrap estimates less than the observed statistic and for the skewness of the bootstrap distribution [21]. We conducted all statistical and spatial analyses using R Statistical Software (version 3.6.1) [22]. The R code is available at https://github.com/sal2222/annual_hsi.

Results

We found that CONUS active-duty Army HSI ambulatory and reportable event rates increased over the study period. Assessing outcome patterns for all types of injuries and illnesses, we observed that ambulatory rates sharply increased over time and hospitalization rates generally declined from 1991 to 1997 and then steadied. Reportable event rates displayed random variability but were the most stable outcome measure over time. The mean counts and rates for each installation are listed in Table 1, along with mean burden, representing the percent of all encounters or events attributed to HSIs. Fourteen installation-outcome type pairs exhibited a positive, linear trend for annual rate at $\alpha = 0.05$ over the included years and two had a negative trend. Fig 1 displays the positive trends of the combined HSI rates from the ten installations over time ($p < 0.001$ for ambulatory and reportable event regression slopes, $p = 0.12$ for hospitalizations).

Table 1. Heat stress illness outcomes (all HSI types).

	**Ambulatory (1998-2018)**			**Hospitalization (1991-2018)**			**Reportable Events (1995-2018)**		
**Installation**	**Mean Count (SD)**	**Mean Rate (SD)**	**Mean Burden % (SD)**	**Mean Count (SD)**	**Mean Rate (SD)**	**Mean Burden % (SD)**	**Mean Count (SD)**	**Mean Rate (SD)**	**Mean Burden % (SD)**
Fort Bliss, TX	28.33 (16.6)	1.57 (0.58) [a]	0.01 (0.00)	1.75 (1.88)	0.11 (0.12)[b]	0.10 (0.09)	3.50 (3.88)	0.22 (0.23)	0.63 (0.68)
Fort Benning, GA	535.48 (290.58)	26.51 (15.00) [a]	0.15 (0.05)	38.00 (20.14)	1.93 (0.96) [a]	2.52 (1.52)	67.38 (54.45)	3.42 (2.84) [a]	18.69 (11.43)
Fort Bragg, NC	702.52 (271.78)	15.51 (5.28) [a]	0.13 (0.05)	31.00 (13.01)	0.72 (0.31)	1.04 (0.53)	140.83 (60.03)	3.21 (1.41)	11.57 (5.51)

Fort Campbell, KY	191.76 (112.63)	6.81 (4.08) [a]	0.05 (0.02)	10.00 (5.48)	0.38 (0.19)	0.54 (0.31)	45.08 (37.7)	1.59 (1.35) [a]	6.77 (6.04)
Fort Hood, TX	110.81 (36.94)	2.68 (1.03) [a]	0.02 (0.01)	7.71 (4.09)	0.19 (0.11) [a]	0.24 (0.15)	27.46 (25.12)	0.64 (0.56)	1.33 (0.90)
Fort Jackson, SC	265.29 (202.53)	27.84 (22.31) [a]	0.13 (0.09)	3.25 (2.88)	0.34 (0.32)	0.38 (0.38)	52.92 (83.18)	5.63 (9.09) [a]	13.22 (17.25)
Fort Leonard Wood, MO	59.86 (51.3)	6.24 (5.50)	0.03 (0.02)	3.21 (2.56)	0.36 (0.29)	0.39 (0.38)	7.00 (5.79)	0.76 (0.65)	3.70 (4.12)
Fort Polk, LA	74.67 (49.06)	9.21 (6.24) [a]	0.06 (0.03)	4.64 (3.01)	0.53 (0.38)	0.72 (0.63)	22.00 (23.8)	2.77 (3.09) [a]	8.35 (7.98)
Fort Riley, KS	42.67 (27.28)	2.89 (1.54)	0.02 (0.01)	1.71 (1.65)	0.12 (0.11) [a]	0.17 (0.16)	8.96 (5.74)	0.67 (0.42)	2.60 (1.94)
Fort Stewart, GA	69.57 (40.98)	4.22 (2.44) [a]	0.03 (0.01)	7.86 (15.67)	0.58 (1.38) [b]	0.57 (0.93)	18.71 (17.68)	1.14 (1.08)	2.88 (2.12)

Rates are per 1,000 persons per year. Burden is calculated as the percentage of HSI encounters compared to the total of all documented injuries and illnesses.

[a] Positive linear regression slope for HSI rate over year at $\alpha = 0.05$.

[b] Negative linear regression slope for HSI rate over year at $\alpha = 0.05$.

Fig 1. Combined HSI outcome rates for ten CONUS Army installations. The line represents a linear model and the shaded area models 95% confidence levels. Note that the scales vary by outcome category by orders of magnitude.

We also detected positive long term (decadal) trends among many indices of heat, compiled over the entire calendar year or restricted to heat season months, across a majority of major CONUS Army installations. Table 2 displays summary statistics for a focused selection of indices and highlights indices with significant positive linear time trends at $\alpha = 0.05$. Among 104 reviewed indices, representing 1,040 index-installation pairs, 599 pairs had significant positive slopes between 1991 – 2018 (57.6%). Note that three of the indices (30 pairs) reflect daily standard deviations and, thus, reflect temperature variability rather than mean temperature. Four index-installation pairs displayed negative slopes: minimum daily indices over the full year at Fort Riley, KS (temperature, WBGT, HI) and Fort Stewart, GA (WBGT).

Table 2. Summary of select annual indices of heat (1991-2018).

	Full Year			Heat Season (May - September)		
**Installation**	**Temperature (°F) Mean (SD)**	**Heat Index (°F) Mean (SD)**	**WBGT (°F) Mean (SD)**	**Temperature (°F) Mean (SD)**	**Heat Index (°F) Mean (SD)**	**WBGT (°F) Mean (SD)**
Fort Bliss, TX	49.97 (1.26) [a]	49.59 (1.29) [a]	48.49 (1.16) [a]	66.14 (1.33) [a]	66.29 (1.52) [a]	64.63 (1.27) [a]
Fort Benning, GA	66.30 (1.08) [a]	67.02 (1.18) [a]	63.43 (1.00) [a]	79.64 (1.39) [a]	82.53 (1.69) [a]	76.15 (0.95) [a]
Fort Bragg, NC	59.02 (1.31) [a]	59.36 (1.39) [a]	56.89 (1.13)	74.82 (1.60) [a]	76.84 (1.94) [a]	72.35 (1.26)
Fort Campbell, KY	62.98 (0.91) [a]	60.75 (0.87) [a]	54.31 (0.80)	75.67 (1.16)	73.98 (1.02)	64.92 (0.74)
Fort Hood, TX	64.92 (1.21) [a]	65.42 (1.26) [a]	62.21 (1.02) [a]	78.89 (1.54) [a]	81.29 (1.71) [a]	75.44 (0.98) [a]
Fort Jackson, SC	55.93 (1.82) [a]	55.59 (1.70) [a]	52.39 (1.28)	74.84 (2.27) [a]	75.49 (2.11) [a]	69.86 (1.22)
Fort Leonard Wood, MO	54.58 (1.08) [a]	52.73 (0.97) [a]	47.98 (0.76)	71.12 (1.48)	69.35 (1.31)	62.42 (0.84)
Fort Polk, LA	66.77 (1.08) [a]	63.84 (1.03) [a]	55.49 (0.76)	82.45 (1.43) [a]	79.29 (1.30) [a]	66.33 (0.91)
Fort Riley, KS	51.50 (1.23)	50.64 (1.23)	50.65 (1.13)	60.93 (1.32)	60.47 (1.44)	60.28 (1.19)
Fort Stewart, GA	63.31 (1.49) [a]	62.75 (1.31) [a]	58.13 (0.96)	80.53 (2.35) [a]	81.05 (1.94) [a]	73.04 (0.90)

[a] Positive linear regression slope at $\alpha = 0.05$, i.e. a warming trend.

In our focused analysis of temperature, HI, and WBGT annual means (Fig 2), we found positive associations with ambulatory visits (rate ratio; RR > 1) at $\alpha = 0.05$ for heat-season temperature and HI, positive associations with hospitalizations for heat-season WBGT, and positive associations with reportable event for full-year temperature and heat-season WBGT. Quantifying our main results, we found that a 1°F increase in mean temperature between May and September is associated with a 1.05 (95% CI: 1.00, 1.11) times greater rate of ambulatory encounters among active-duty Army soldiers at CONUS locations, controlling for installation-specific effects. The same temperature increase was associated with an increase in

hospitalization rates by a factor of 1.14 (95% CI: 1.06, 1.21) and a marginal increase in reportable event rates by a factor of 1.10 (95% CI: 0.98, 1.23).

Fig 2. Rate ratios for full−year and heat season indices of heat and HSI encounters at 10 CONUS U.S. Army installations. RRs per 1 degree increase in annual index of heat (mean of daily means) from 2−year block bootstrap negative binomial models with basic (empirical) confidence intervals based on 10,000 replicates, controlling for installation−level effects. Solid points reflect the mean of bootstrap estimates and unfilled points reflect the original sample (non−bootstrap) estimate.

Out of 312 assessed index-outcome pairs, 142 exhibited positive associations after controlling for installation-level effects and time trends (Table 3). WBGT and HI indices were more likely to indicate a positive association than temperature-only indices. Indices averaged over the full calendar year displayed a higher proportion of positive associations than those averaged over heat season months. Indices based on hourly counts above threshold values were more likely to show positive associations than indices based on counts of days above thresholds. We observed similar associations between anomaly based (relative) and non-anomaly based (absolute) indices.

Table 3. Annual scale index-HSI outcome rate ratio 95% confidence interval positions from 2-year block bootstrap negative binomial models.

	**Positive RR >1 (N=142)**	**Null RR = 1 (N=148)**	**Negative RR < 1 (N=22)**	**Total (N=312)**
**Outcome Type**				
Ambulatory	66 (63.5%)	37 (35.6%)	1 (1.0%)	104

Hospitalizations	22 (21.2%)	64 (61.5%)	18 (17.3%)	104
Reportable Events	54 (51.9%)	47 (45.2%)	3 (2.9%)	104
**Index Type**				
Temperature	29 (37.2%)	44 (56.4%)	5 (6.4%)	78
Heat Index	55 (48.2%)	48 (42.1%)	11 (9.6%)	114
WBGT	58 (48.3%)	56 (46.7%)	6 (5.0%)	120
**Timeframe**				
Full Year	83 (50.3%)	73 (44.2%)	9 (5.5%)	165
May-Sep	59 (40.1%)	75 (51.0%)	13 (8.8%)	147
**Exposure Measure**				
Degree-based	38 (42.2%)	45 (50.0%)	7 (7.8%)	90
Day-based	81 (45.0%)	87 (48.3%)	12 (6.7%)	180
Hour-based	23 (54.8%)	16 (38.1%)	3 (7.1%)	42
**Anomaly-Based**				
No	60 (46.5%)	60 (46.5%)	9 (7.0%)	129
Yes	82 (44.8%)	88 (48.1%)	13 (7.1%)	183

Counts (row-wise percentage) from basic (empirical) confidence intervals, controlled for location-level effects.

In our sensitivity analyses of various models, non-bootstrap negative binomial models adjusted for year returned RR estimates closer to the null than 2-year block bootstrap models. Results from standard bootstrap models (single year replacement) approximated negative binomial models without adjustment for year. 3-year block bootstrap models returned wider CIs than 2-year block models, with mean estimates shifted in both directions.

Discussion

In this study, we identified positive decadal trends among indices of heat and humidity and among HSI outcomes at active-duty CONUS Army installations. We found overall positive association patterns for ambulatory and reportable event outcomes with temperature, HI, and WBGT indices in absolute and relative measures. The largely null finding for hospitalization associations may be due to the low number of annual HSI admissions at many locations. Five of the ten CONUS installations averaged fewer than five HSI hospitalizations per year (Table 1). Considering the relative rarity of diagnosed HSI hospitalizations, the availability of ambulatory encounter and reportable event data adds substantial value for the characterization of HSI morbidity.

We evaluated multiple combinations of heat and humidity index characteristics (104 indices, 312 total index-outcome pairs) in our negative binomial association model and classified the resulting RRs as positive, null, or negative. These findings do not necessarily indicate that sets of indices are more correct than others; rather, they may be more sensitive to detecting associations at an annual scale in support of our hypotheses that heat indices and HSI outcomes are related. There is no mechanism for a negative association and we recognize that some associations in either direction may be due to chance. Overall, the finding that WBGT and HI indices “outperformed” ambient temperature indices was expected because they capture the effect of humidity. It was unexpected, however, that indices averaged over the full year would display a positive association rate greater than those averaged over a defined heat season. This result furthers evidence for expanding the boundaries of the traditional heat season and incorporating prevention efforts throughout the year. Among exposure measure types, indices

based on counts of hours were more sensitive to a positive association than day-based or degree-based indices, reflecting a strength of the high-resolution NLDAS-2 dataset.

In these analyses, we assumed that the frequencies and intensities of outdoor training events remained consistent over time for each location and that population-level risk factors did not fluctuate. We made these assumptions considering that the major unit compositions and Training and Doctrine Command (TRADOC) and Forces Command (FORSCOM) mission sets at the selected sites remained mostly stable over the evaluated timeframe. Challenges to this assumption could occur from installation population changes due to extended large unit overseas deployments and organizational changes, such as the movement of the Armor School from Fort Knox, KY to Fort Benning, GA in 2011. Likewise, demographics of age, sex, and ethnicity among the base population active duty soldiers have not markedly changed, although generational change in overall fitness levels and body composition represent a risk factor of concern [23–25]. Additionally, we assumed that HSI prevention measures, including annual safety training requirements and monitoring of WBGT heat categories with associated work-rest cycle and hydration recommendations, had not meaningfully varied over the study time-course [3]. The block bootstrap process to adjust for time trends, along with the inclusion of installation indicator variables, mitigate these potential changes within and between installations over time.

There is also a need to consider whether other time-varying trends account for changes in reported HSI rates. Changes in access to care, case definitions, and reporting systems and procedures can all contribute to long-term trends in the outcomes we studied. We observed impacts from such changes when comparing the rates of all ICD-coded illnesses and injuries over time, especially for ambulatory rates. The block bootstrap method also controls for this serial correlation in outcomes. Another limitation with our annually aggregated health outcome

counts is that we were unable to discern incident cases from follow-up encounters. The ambulatory counts and rates are therefore elevated above incidence-based case definition levels; however, in this aspect they provide representation of the overall burden on the healthcare system from HSIs.

This study assesses the long-term impacts of environmental changes on direct heat-related morbidity; however, it lacks the within-year temporal resolution needed to inform day-to-day or operational level decisions. Important short-term exposure parameters include the intensity, duration, and timing in season of extreme heat events [26]. Further study of HSI morbidity among physically active populations with outdoor environmental exposures could examine the short-term exposure-response relationship between heat and humidity indices and daily outcomes, considering lagged and non-linear effects and controlling for individual-level risk factors.

Conclusion

U.S. Army CONUS installations have broadly experienced rising temperature conditions and increased rates of HSI morbidity over the past two to three decades. In this study, we determine that temperature, HI, and WBGT indices are positively associated with rates of ambulatory encounters and reportable events, controlling for installation-levels effects and accounting for potential confounding by long-term trends in the outcomes and exposures. The annual-scaled rate ratios and their uncertainties can be applied to climate projections for a wide range of thermal indices to estimate future HSI burden and impacts to medical readiness. As an example, we obtained a RR of 1.05 for ambulatory HSI rates for each °F increase in mean temperature between May and September. In 2018, the active-duty population of approximately

204,291 at the included ten CONUS installations reported 3,612 ambulatory HSI encounters. Applying our effect estimate, with a 1 °F increase in the heat season mean temperature, we project an increase to 3,793 HSI ambulatory encounters (+181 cases) in the absence of additional adaptations or control measures.

References

1. Armed Forces Health Surveillance Branch. Update: Heat illness, active component, U.S. Armed Forces, 2017. MSMR. 2018;25: 6–12.
2. Schickele E. Environment and Fatal Heat Stroke: An Analysis of 157 Cases Occurring in the Army in the U. S. During World War II. Mil Surg. 1947;100: 235–256. doi:10.1093/milmed/100.3.235
3. Headquarters, Department of the Army and Air Force. Heat Stress Control and Heat Casualty Management (TB MED 507/AFPAM 48-152(I)). Washington, D.C.; 2003 Mar. Report No.: TB MED 507/AFPAM 48-152(I).
4. U.S. Army Public Health Center. 2019 Health of the Force. 2020. Available: https://phc.amedd.army.mil/topics/campaigns/hof/Pages/default.aspx
5. U.S. Global Change Research Program. Climate Science Special Report: Fourth National Climate Assessment, Volume I. U.S. Global Change Research Program, Washington, DC; 2017. Available: https://science2017.globalchange.gov/
6. Macpherson RK. The Assessment of the Thermal Environment. A Review. Br J Ind Med. 1962;19: 151–164.
7. Budd GM. Wet-bulb globe temperature (WBGT)—its history and its limitations. Journal of Science and Medicine in Sport. 2008;11: 20–32. doi:10.1016/j.jsams.2007.07.003
8. Anderson GB, Bell ML, Peng RD. Methods to Calculate the Heat Index as an Exposure Metric in Environmental Health Research. Environ Health Perspect. 2013;121: 1111–1119. doi:10.1289/ehp.1206273
9. DeFraites RF. The Armed Forces Health Surveillance Center: enhancing the Military Health System's public health capabilities. BMC Public Health. 2011;11 Suppl 2: S1. doi:10.1186/1471-2458-11-S2-S1
10. World Health Organization, editor. The International Classification of Diseases, 9th Revision, Clinical Modification. 9th revision, 6th ed. Geneva: World Health Organization; 2011.

11. World Health Organization, editor. International Statistical Classification of Diseases and Related Health Problems. 10th revision. Geneva: World Health Organization; 2019. Available: https://icd.who.int/browse10/2019/en

12. Xia Y, Mitchell K, Ek M, Sheffield J, Cosgrove B, Wood E, et al. Continental-scale water and energy flux analysis and validation for the North American Land Data Assimilation System project phase 2 (NLDAS-2): 1. Intercomparison and application of model products. Journal of Geophysical Research: Atmospheres. 2012;117. doi:10.1029/2011JD016048

13. Military Installations, Ranges, and Training Areas - Data.gov. 18 Jan 2017 [cited 24 Jan 2021]. Available: https://catalog.data.gov/dataset/military-installations-ranges-and-training-areas

14. Liljegren JC, Carhart RA, Lawday P, Tschopp S, Sharp R. Modeling the wet bulb globe temperature using standard meteorological measurements. J Occup Environ Hyg. 2008;5: 645–655. doi:10.1080/15459620802310770

15. Lemke B, Kjellstrom T. Calculating Workplace WBGT from Meteorological Data: A Tool for Climate Change Assessment. Industrial Health. 2012;50: 267–278. doi:10.2486/indhealth.MS1352

16. DeGroot D, Martin R. Within-year Exertional Heat Illness Incidence in U.S. Army Soldiers, 2008-2012: Fort Belvoir, VA: Defense Technical Information Center; 2015 Jun. doi:10.21236/ADA621280

17. Venables WN, Ripley BD. Modern Applied Statistics with S. 4th ed. New York: Springer-Verlag; 2002. doi:10.1007/978-0-387-21706-2

18. Önöz B, Bayazit M. Block bootstrap for Mann–Kendall trend test of serially dependent data. Hydrological Processes. 2012;26: 3552–3560. doi:10.1002/hyp.8438

19. Canty A, Ripley BD. boot: Bootstrap R (S-Plus) Functions. 2019.

20. Davison AC, Hinkley DV. Bootstrap Methods and Their Applications. Cambridge: Cambridge University Press; 1997. Available: http://statwww.epfl.ch/davison/BMA/

21. Efron B. Better Bootstrap Confidence Intervals. Journal of the American Statistical Association. 1987;82: 171–185. doi:10.1080/01621459.1987.10478410

22. R Core Team. R: A Language and Environment for Statistical Computing. Vienna, Austria: R Foundation for Statistical Computing; 2019. Available: https://www.R-project.org/

23. Bedno SA, Li Y, Han W, Cowan DN, Scott CT, Cavicchia MA, et al. Exertional heat illness among overweight U.S. Army recruits in basic training. Aviat Space Environ Med. 2010;81: 107–111.

24. Williams VF, Oh G-T, Stahlman S. Incidence and prevalence of the metabolic syndrome using ICD-9 and ICD-10 diagnostic codes, active component, U.S. Armed Forces, 2002-2017. MSMR. 2018;25: 20–25.

25. Reyes-Guzman CM, Bray RM, Forman-Hoffman VL, Williams J. Overweight and obesity trends among active duty military personnel: a 13-year perspective. Am J Prev Med. 2015;48: 145–153. doi:10.1016/j.amepre.2014.08.033

26. Anderson GB, Bell ML. Heat waves in the United States: mortality risk during heat waves and effect modification by heat wave characteristics in 43 U.S. communities. Environ Health Perspect. 2011;119: 210–218. doi:10.1289/ehp.1002313

Fig 1

Click here to access/download;Figure;figure1.tif

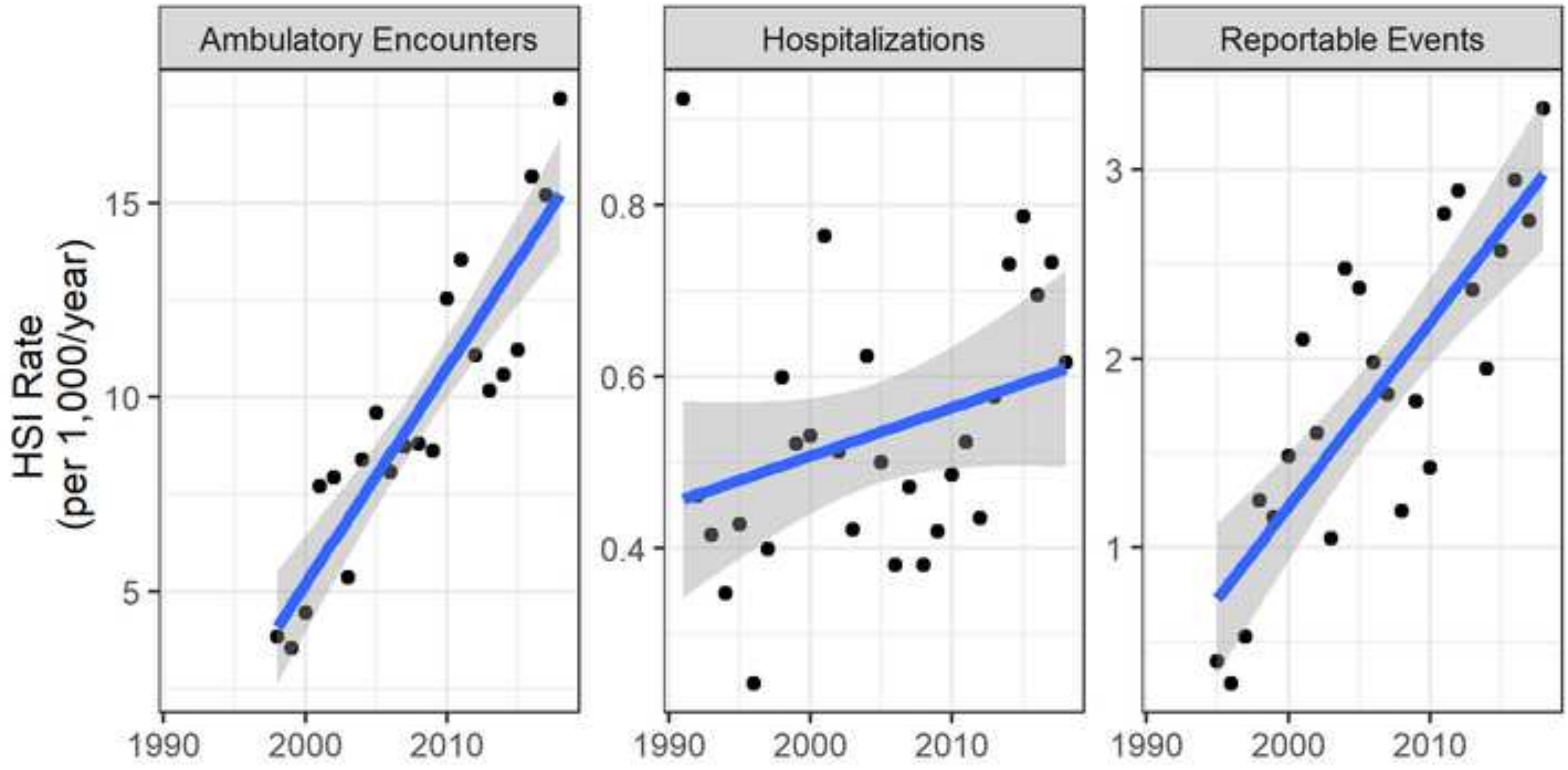

Fig 2

Click here to access/download;Figure;figure2.tif

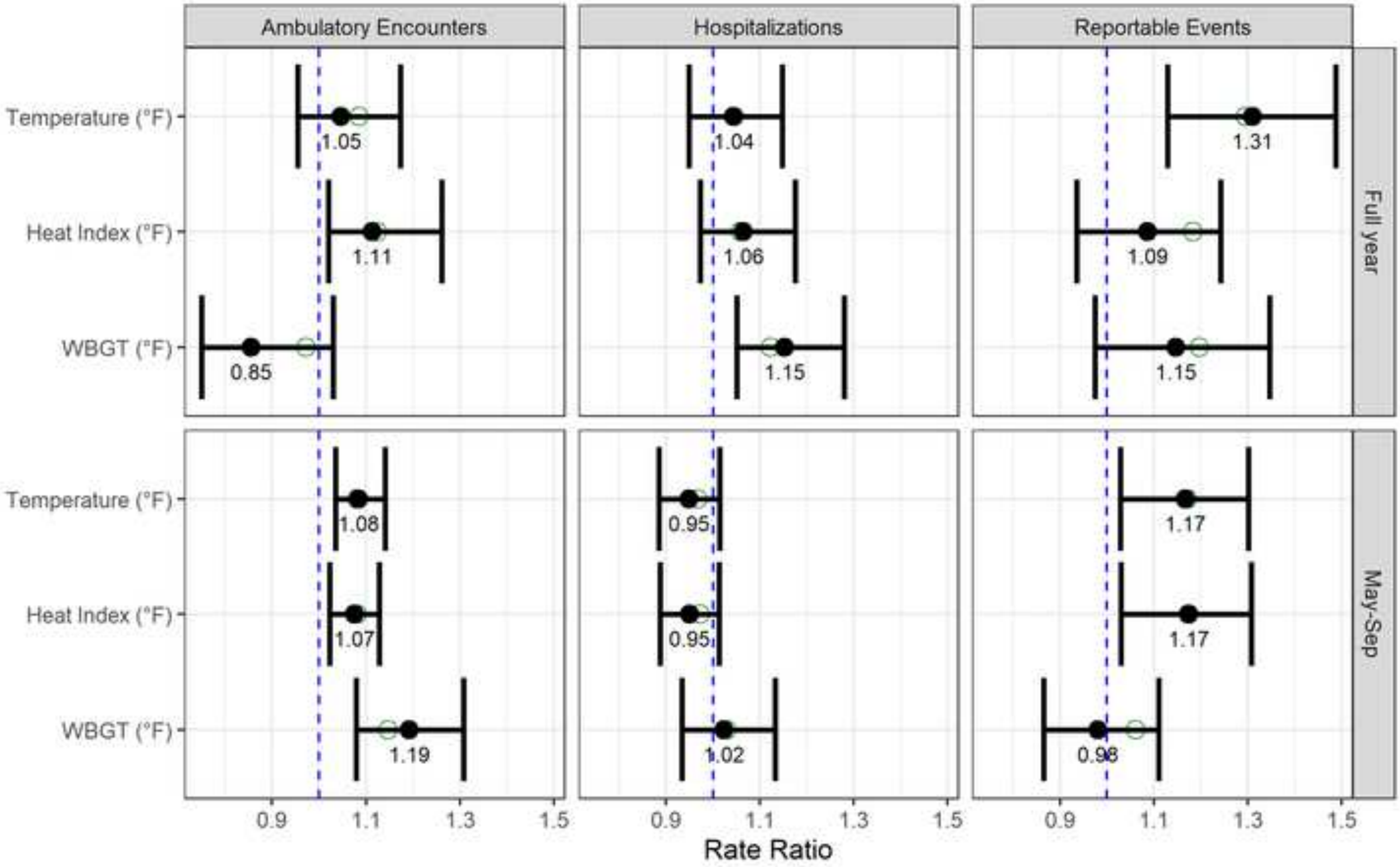

S1 Table

Click here to access/download
Supporting Information
S1 Table.docx

S2 Table

Click here to access/download
Supporting Information
S2 Table.docx
